# Characterization of the Detailed Fatty Acid Profiles of a Large Number of Types of Cheese from the Mountains and Plains

**DOI:** 10.3390/foods13244040

**Published:** 2024-12-14

**Authors:** Giovanni Bittante, Nicolò Amalfitano, Franco Tagliapietra, Stefano Schiavon, Claudio Cipolat-Gotet, Giorgia Stocco

**Affiliations:** 1 Department of Agronomy, Food, Natural Resources, Animals and Environment, University of Padova, 35020 Padua, Italy; bittante@unipd.it (G.B.); franco.tagliapietra@unipd.it (F.T.); stefano.schiavon@unipd.it (S.S.); 2 Department of Veterinary Science, University of Parma, 43126 Parma, Italy; claudio.cipolatgotet@unipr.it (C.C.-G.); giorgia.stocco@unipr.it (G.S.)

**Keywords:** CLA, omega-3, Alpine mountain cheeses, goat cheeses, PDO cheeses, pasta filata cheeses, healthy foods, cheese nutritional value

## Abstract

The aim of this study was to characterize the highly detailed fatty acid (FA) profiles of 258 cheeses of 18 different categories of cheese collected in the mountains and on the plains of the Veneto region (Italy). The results clearly showed that, aside from the distinctive FA profiles of goat cheeses (more short-chain FAs and fewer MUFAs), the three categories of Formaggio di Malga (artisanal cheeses produced on temporary summer farms on Alpine pastures where transhumance is practiced) were very different from the other cheese categories in terms of their much higher CLA and omega-3 contents. Two categories of cheese from permanent farms in the mountains (Morlacco del Grappa and Monte Veronese PDO) were intermediate, and two other categories of cheeses originating in the mountains (Asiago PDO and Montasio PDO), but now produced mainly on the plains, were not distinguishable from the other cheese categories. The very detailed profile (65 individual FA, 11 isomers, and 12 groups of FAs) and the large number of cheese types analyzed (18) may represent a useful reference for future investigations, especially on the causes of variability in FAs and on their relationships with sensory properties and nutrition/health in humans.

## 1. Introduction

There is currently great interest in the fatty acid profiles of milk and dairy products, especially with respect to the implications of their consumption on human health.

New findings have recently intensified the debate over the impact of dietary fatty acids on human health [1]. Large meta-analyses have found either no relationships between saturated fatty acids and cardiovascular diseases (CVDs) or inconsistent results [2]. Furthermore, the value of large categories of fatty acids, like saturated, unsaturated, omega-3, trans-fatty acids, etc., have also been questioned in light of emerging scientific evidence showing that, within each category, different fatty acids have very different health impacts [3], as do molecules present in low concentrations. Highly detailed fatty acid profiles of different foods are therefore needed. A state-of-the-art review in the *Journal of the American College of Cardiology* concluded that the most recent meta-analyses of randomized trials and observational studies had found that reducing SFA intake had no beneficial effects on CVDs and total mortality and conversely found protective effects against stroke [4].

The concept of an individual nutrient as a useful tool for describing the relationships between foods and human health has also been questioned. In fact, the authors of the aforementioned review concluded that “It is also apparent that the health effects of foods cannot be predicted by their content in any nutrient group without considering the overall macronutrient distribution” [4]. Furthermore, “Whole-fat dairy, unprocessed meat, and dark chocolate are SFA-rich foods with a complex matrix that are not associated with increased risk of CVD. The totality of available evidence does not support further limiting the intake of such foods” [4]. The need to re-evaluate dietary recommendations regarding saturated fatty acids has also been highlighted by other studies [5,6]. The importance of the interactions between the different nutrients and of the matrix in determining the health effects of foods, particularly cheese, is well documented [7]. However, there are many different types of cheese, which may be made from milk of different dairy species in different geographical areas and farming systems, processed using a variety of procedures (milk standardization, culture addition, heating, enzyme addition, curd cooking, pressing, salting, etc.), and ripened for widely varying lengths of time and under varying conditions [8,9,10]. It is well known that the fatty acid profile of cheese is not identical to that of the milk from which it is made due to the different recovery rates of the different fatty acids in the curd and the modifications induced by native and added enzymes and microflora [11].

A search of scientific articles in international databases using the keywords fatty acid and milk returned almost 25,000 references, about one-sixth of which included these keywords in the articles’ titles. Far fewer articles dealt with the fatty acid profiles of cheese (slightly more than 3000, with fewer than 500 with this keyword in the title, and no reviews). Where the keywords included specific cheeses or cheese types, the number of articles decreased to a few dozen, with a very small number comparing different types of commercial cheeses [12,13,14,15,16]. Moreover, the number of fatty acids analyzed is generally small (between 10 and 20), and the comparisons often concern the large fatty acid categories.

In order to better assess the variability in the nutritional and health attributes of different types of cheeses, a large project, “Caseus Veneti”, was established in the Veneto region (northeastern Italy) with the aim of characterizing at least 1000 different cheeses of at least three dozen different cheese types produced by more than 100 cheese factories. The project is described in detail in a previous study [17]. Having studied the latent factors explaining most of the variability in the physical and chemical characteristics of the cheeses [18] and their relationships with vibrational spectra as a tool for rapidly predicting the chemical composition of cheese [19,20], the aim of the present study was to obtain a highly detailed fatty acid profile of many different types of cheeses that could be used as a reference for further studies, especially in relation to human nutrition and health. In particular, to quantify the variability of the FA profile, we compared cheeses from different dairy species (bovine and caprine), different production areas and farming systems (mountains *vs* plains), traditionally protected cheeses *vs* modern commercial products, and cheeses with very different ripening times.

## 2. Materials and Methods

### 2.1. Origin of the Cheeses Analyzed

A total of 1050 cheeses from 37 different cheese types were sampled over the three years [17] of the Caseus veneti, an annual cheese exhibition and competition (today simply known as “Caseus” https://caseusitaly.it/, accessed on 1 October 2024), sponsored by the Veneto Regional Government (northeastern Italy). The most important aspects of cheese-making technology for every cheese type were recorded and summarized in the previous study [17]. For every individual cheese, data on physical (weight, diameter, and height of the wheels, or length, width, and height of the blocks) and chemical (moisture/total solids, fat, protein, ask and water-soluble nitrogen) characteristics were obtained [17]. Information on dairy farms and animals was not available.

Of all the sampled cheeses, 234 were selected to determine their detailed fatty acid profiles. The number of selected cheeses was proportional to the number of individual cheeses of each cheese type that were available, and always included the three highest-ranking cheeses of each cheese type. As the cheeses produced on Alpine summer farms are of special interest, a larger proportion of these was selected from among the two Caseus veneti Malga cheeses. A further 24 Malga cheeses from competitions in the nearby Autonomous Province of Trento were also analyzed (258 cheeses analyzed in total).

### 2.2. Categorization of the Cheeses Analyzed

Given the modest number of cheeses selected per cheese type, those with similar characteristics were combined to form 18 categories. The main characteristics of each category and the number of cheese types included therein are summarized in Table 1.

Cheese types with Protected Designation of Origin (PDO) status [22] included seven categories:

- Asiago PDO cheese category comprised four PDO cheese types differentiated mainly by the length of ripening: Asiago Fresco (full fat); Asiago Stagionato Mezzano, Asiago Stagionato Vecchio, and Asiago Stagionato Stravecchio, all made from partly skimmed milk; these cheeses are originally from the Altopiano di Asiago in the Prealps of the Veneto region, although the area of production also includes the foothills and plains below the mountains;
- Casatella Trevigiana PDO cheese and commercial rindless fresh (freschi) and very fresh (freschissimi) cheeses; these cheeses are mainly produced in the plains of Treviso province in the Veneto region;
- Grana Padano PDO category comprises two cheese types (12–20 and ≥20 months of ripening); the cheeses analyzed were produced mainly on the plains of the Veneto region, although the PDO area also includes parts of the regions of Lombardy, Emilia-Romagna, and Piedmont;
- Montasio PDO category comprising three PDO cheese types differing mainly in the length of ripening: Montasio Fresco, Montasio Mezzano, and Montasio Stagionato; these cheeses were originally from the Carnic Alps in the nearby region of Friuli-Venezia Giulia, although the production area also includes the hills and plains of the eastern Veneto region;
- Monte Veronese PDO category comprises three PDO cheese types differing mainly in the length of ripening: Monte Veronese latte intero (full fat), Monte Veronese d’Allevo mezzano, and Monte Veronese d’Allevo vecchio, from partly-skimmed milk; these cheeses are produced mainly in the mountains and hills of Verona province (western Veneto region);
- Piave PDO cheese and other commercial hard cheese types. Piave is produced in the upper Piave valley in the eastern Veneto region, while the other commercial hard cheeses are produced in various provinces of the Veneto region;
- Provolone PDO cheese and other commercial ripened *pasta filata* cheeses. There are two types of Provolone PDO cheeses with different ripening lengths: Provolone Dolce and Provolone Piccante; Provolone cheese comes originally from the plains of the nearby Lombardy and Piedmont regions, and in the Veneto region it is produced mainly in the western province of Verona, while other commercially ripened *pasta filata* cheeses are produced in different areas in the region, mainly on the plains;

One category comprises a cheese type with Traditional Specialty Guaranteed (TSG) designation in accordance with European Union regulations [21,22] and a type that includes other soft pasta filata cheeses:

- Mozzarella TSG and other commercial fresh pasta filata cheeses produced mainly on the plains of the Veneto region.

Four categories, each comprising a single cheese type designated as a Traditional Veneto Cheese (RTC) by the Veneto region, and one category comprising a cheese type assigned a similar designation by the Autonomous Province of Trento (Trentino di Malga):

- Formaggio imbriago RTC is produced mainly on the plains of the eastern Veneto region; “imbriago” means “drunk”, with the cheese being ripened in marc, must, or wine;
- Morlacco del Grappa RTC is produced in the central Prealps of the Veneto region (the Grappa massif) and its foothills;
- Malga fresco RTC is made from milk produced exclusively on Alpine summer farms in the Veneto mountains, often in small dairies on the *malga* (temporary summer highland farm) according to traditional artisanal procedures and ripened for 2–6 months;
- Malga vecchio RTC has the same origin as the previous cheese category but is ripened for 12 months or more;
- Trentino di Malga is produced in a similar way to the previous two categories of cheese, but *malga* cheeses produced in the Autonomous Province of Trento have variable ripening lengths;

Lastly, five categories comprise exclusively commercial types of cheeses produced in the Veneto Region, mainly on the plains:

- Caciotta and Latteria are two types of soft and semi-soft cheeses differentiated in particular by the weight of the wheels (<1.0 kg and >1.0 kg, respectively);
- Flavored cheeses is a category comprising five different types of cheeses obtained by adding ingredients other than rennet and salt to the milk during cheese making or ripening: Formaggi al pepe or peperoncino, with the addition, respectively, of pepper or chili; Formaggi con erbe, fieno o spezie, with the addition of herbs, hay, or spices; Formaggi affumicati are smoked cheeses; Formaggi erborinati are blue cheeses; and Formaggi alla birra o balsamico are manufactured via the addition of beer or balsamic vinegar; the cheese-making procedures, wheel weights, ripening lengths, and conditions are highly variable;
- Semi-hard cheeses are made following procedures similar to those for the PDO cheeses of Alpine origin with an intermediate ripening length but are produced outside the areas specific to the various PDO denominations or without adhering to all the production regulations of the PDO Consortia; these cheeses are produced in various areas of the Veneto region;
- The Treated rind category comprises two cheese types: Crosta fiorita with a bloomy rind and Crosta lavata with a washed rind; these cheeses are produced in various areas of the Veneto region;
- The Goat cheese category comprises two cheese types: Caprino a coagulazione acida, with acid milk coagulation, and Caprino a coagulazione presamica, with rennet coagulation; the majority of these cheeses are produced in the hills and mountains of the Veneto Region.

### 2.3. Sampling and Storing Cheeses

The cheese wheels or blocks were halved and a representative central slice of at least 300 g was taken, weighed, immediately vacuum-packed, and refrigerated at +4 °C, then transported to the Milk Quality Laboratory of the Department of Agronomy, Food, Natural Resources, Animals and Environment (DAFNAE) of the University of Padova (Legnaro, Italy). Within 3 d of sampling, the packaged samples were opened, the rind was removed, and a sub-sample (>100 g) was ground, frozen at −80 °C, and stored till the analysis.

### 2.4. Lipid Extraction and Esterification

Lipids were extracted using a Soxtec extraction apparatus (ST 255; Foss Electric, Foss A/S, Hillerød, Denmark) with pentane as a solvent according to the ISO methodology [23]. Each sample was thawed and an amount containing approximately 4 g of fat (>10 g) was transferred into a mortar and ground with a 1 + 1 mixture of sand and sodium sulfate to yield a dry sample. The extraction was performed with 50 mL of pentane at 35 °C for a total duration of 3 h. Pentane was then evaporated under a stream of nitrogen at 50 °C to obtain the pure fat sample. Forty milligrams of the pure fat was sampled and subjected to acid–base transesterification and methylation, as described by Jenkins [24]. Specifically, 2 mL of sodium methoxide (0.5 M in methanol) and 2 mL of hexane were added to the sample, and the mixture was incubated at 50 °C for 10 min. After cooling to room temperature, 3 mL of hydrochloric acid in methanol was added and the sample was heated to 80 °C for 10 min. Subsequently, 2 mL of hexane and 3 mL of K_2_CO_3_ solution (0.43 M) were added to the cooled sample. After centrifugation at 400 g and 4 °C for 5 min, the organic phase was transferred to a GC vial for analysis.

### 2.5. Two-Dimensional Gas Chromatography Analysis

Highly detailed fatty acid profiles of the samples were obtained using GC × GC equipment (Agilent 7890A, Agilent Technologies, Santa Clara, CA, USA) using two columns in series with a modulator (G3486A CFT, Agilent Technologies), and with an automatic sampler (7693, Agilent Technologies) and flame ionization detector connected to the chromatography data system software (Agilent Chem Station v. C.01.10, Agilent Technologies). The operating conditions, reported in detail in Schiavon et al. [25], were as follows: first column of 75 m × 180 μm (internal diameter) × 0.14 μm film thickness (23348U; Supelco, Bellefonte, PA, USA), H_2_ carrier flow of 0.22 mL/min; second column of 3.8 m × 250 μm (internal diameter) × 0.25 μm film thickness (J&W 19091-L431; Agilent Technologies, Santa Clara, California, U.S.), H_2_ carrier flow of 22 mL/min. The oven temperature was set to 50 °C (held for 2 min), increased to 150 °C at a rate of 50 °C/min (held for 15 min), then increased to 240 °C at 2 °C/min (held for 84 min). Valves were as follows: modulation delay, 1 min; modulation period, 2.90 s; sample time, 2.77 s. Gas flows were as follows: hydrogen, 20 mL/min; air, 450 mL/min. A 1 μL sample volume was injected at a split ratio of 150:1. The resulting two-dimensional chromatograms were analyzed with comprehensive GC × GC software (GC Image Software v. 2.2, Zoex Corp., Houston, TX, USA) to calculate the cone volume of each FA.

### 2.6. Identification and Quantification of FAs

The individual FAs were identified using two methods. The first involved comparing the cone positions in the chromatogram with those obtained from various GC reference standards containing a mixture of pure FAs. These reference standards were #674 and #463 (Nu-Chek Prep, Elysian, MN, USA) plus 5 CLAs: 18:2cis-9, trans-11 (#UC-60M; Nu-Chek Prep, Elysian, MN, USA), 18:2trans-10, cis-12 (#UC-61M; Nu-Chek Prep, Elysian, MN, USA), 18:2cis-9, cis-11 (#1256; Matreya LLC., Pleasant Gap, PA, USA), 18:2trans-9, trans-11 (#1257; Matreya LLC, Pleasant Gap, PA, USA), and 18:2cis-11, trans-13 (#1259; Matreya LLC, Pleasant Gap, PA, USA). Additional FAs were identified by comparing the elution order with the position of each FA in the 2-dimensional chromatogram produced by the comprehensive GC × GC software (GC Image Software, Zoex Corp.). Each FA was quantified as the cone volume of each FA peak as a percentage of the volume of all FAs. The FAs identified using the standards underwent separate statistical analyses, whereas the MUFAs and PUFAs identified by position were summed into groups according to the length of their carbon chain and degree of unsaturation.

### 2.7. Statistical Analysis

The individual fatty acids (g/g total FA × 100) and the various related groups were analyzed using the following linear mixed model in SAS (https://www.sas.com/en_in/home.html, accessed on 1 December 2024, SAS Inst. Inc., Cary, NC, USA):

y_ij_ = µ + cheese-type_i_ + e_ij_,

where y_ij_ = the fatty acid traits (65 individual FAs, 11 sums of FA isomers, 12 groups of FAs); μ = the overall intercept of the model; cheese-type_i_ = the fixed effect of cheese type (1 to 18); and e_ij_ = the residual random error~N (0, σ2e).

To avoid calculating and presenting 153 possible multiple comparisons between the 18 cheese types for every FA trait, the significance of the solutions of each cheese type (i.e., the significance of the difference between a given cheese type and the general mean of all cheese types) was presented. For each fatty acid trait, a single cheese category was considered significantly different from the average if its solution differed from 0.00 at *p* < 0.05.

Multivariate Principal Component Analysis (PCA) was performed using Statistica 7.1 (StatSoft, Paris, France) to explore the relationships among the FA profiles of the 18 cheese types.

## 3. Results and Discussion

### 3.1. Composition and Detailed Fatty Acid Profiles of Cheeses

The chemical composition and physical characteristics of all the cheeses analyzed (1050), from which the 258 reported in this study were selected, were reported and discussed in detail in a previous study from the same project [17] and are therefore not discussed here. Only data on the total solids and lipid contents of cheeses used in this study are summarized in Table 2 to favor the comprehension of data on fatty acid profiles.

The 18 categories of cheeses have large differences in their contents of total solids (Table 2).

Six categories had significantly lower than average solid contents (*p* < 0.05), with least squares mean (LSM) values in the range of 40.4 to 54.7%, and higher than average moisture contents (45.3 to 59.6%): Casatella Trevigiana + other fresh cheeses, Mozzarella TSG and other soft *pasta filata* cheeses, Morlacco del Grappa (RTC), and the commercial cheese categories goat cheeses, Treated rind cheeses, and Caciotta and Latteria. All the other cheese categories had high contents (*p* < 0.05) of total solids (64.3 to 71.4%), except for the Provolone PDO + other firm *pasta filata* cheese category and the Malga fresco RTC category, which had an intermediate solids content (61%).

The lipid content was less variable (Table 2). Only two categories had a higher (*p* < 0.05) than average fat content: Piave PDO + other hard cheeses (34.54%) and the semi-hard cheeses (33.63%), which were also the categories with cheeses with the highest contents of total solids. Three categories of fresh cheeses had the lowest (*p* < 0.05) fat content: Mozzarella TSG + other soft *pasta filata* cheeses (19.93%), Morlacco del Grappa RTC (23.14%), and goat cheeses (19.83%). The large variability in the lipid contents of different cheeses could mask the smaller variability in the proportion of individual fatty acids in their sum. Thus, it is expected that the cheeses characterized by the highest level of lipids will also be those with the highest levels of total and individual fatty acids. This means that the variability in the quantity of lipids can mask the variability in the quality of lipids. This is the reason why the individual fatty acids and their groups have been reported and discussed only in terms of their proportion to the sum of all the fatty acids of the cheese sample analyzed (fatty acid profile).

The results showed that cheese has a very complex fatty acid profile. We have reported here the proportions of 65 individual fatty acids, several dozen other isomers summed for 11 fatty acids, and 12 groups of fatty acids. With few exceptions [12], the large majority of scientific articles report the proportions of only one to two dozen fatty acids [14,15,16,26,27,28], or a few fatty acid categories [13,29,30,31]. The detail obtained in this study was made possible through the use of 2-dimensional gas-chromatography, also used in other studies [32,33]. The analysis of many fatty acids has become increasingly important because of the growing scientific evidence concerning the effects of individual fatty acids on human metabolism and health [3,5]. Given the role of these substances as not only nutrients but also bio-active compounds with enzymatic/hormonal-type action, it is important to also quantify those fatty acids that are present in foods in low or very low amounts [34]. Moreover, the fundamental role of milk fatty acids and their enzymatic and microbiological metabolism on the development of sensory properties of cheese are well known [35].

In our previous analyses of fatty acid profiles carried out with the same instrument, we were able to identify and quantify between 51 and 56 individual fatty acids in milk [36,37] and 56 in cheese [36]. It is obvious that there is a direct relationship between the fatty acid profiles of unprocessed milk and the dairy products derived from it, but there are also some differences. In research aimed at studying the sequential evolution of the fatty acid profiles of milk and dairy products and by-products during the cheese-making process, we analyzed 11 dairy matrices obtained during seven cheese-making sessions [36]. The fatty acid profiles of milk often differ between the morning and evening milkings, especially in the proportions of some SFAs and MUFAs [36]. Furthermore, where the milk is partially skimmed to produce some types of cheese, differential migration gives rise to differences between the fatty acid profiles of the partly skimmed milk and the cream, especially when they are separated by natural creaming [36], and also between the cream and the fresh cheese. Other changes were observed during the storing, heating, and culturing of the milk in the dairy so that the milk in the vat, before the addition of rennet, could be differentiated from the milk at the farm gate [36].

Although about 80 to 90% of milk fat is recovered in the curd used for shaping the cheese wheels during cheese-making, the individual fatty acids are not recovered at the same rate, nor is their recovery equal to the recovery of the milk fat. These differences are affected by the cow’s diet [25]. Relevant differences have been found for different fatty acids [25]. For example, there are some differences between the fatty acid profiles of whey and fresh cheese. Larger differences were found between ricotta (made from the whey) and *scotta* (the residual liquid from this process) [36]. Lastly, in a previous study on Malga cheese, we monitored the fatty acid profiles of the cheeses during ripening and found large differences between the fresh cheese and the same cheese after 6 months of ripening, but more moderate differences between the cheeses after 6 and 12 months of ripening [36]. These alterations seem to be mainly the result of microbial activity during ripening and regard particularly the MUFAs present in small concentrations [25,27]. The differences in the fatty acid profiles of different cheeses cannot, therefore, be only due to differences in the fatty acid profiles of the milk they are derived from but also reflect differences in milk storage and culturing, cheese-making procedures, and ripening length and conditions.

As in the scientific literature, there is a lack of studies reporting detailed fatty acid profiles of many categories of cheeses. To make available a reference for future studies and for nutritional utilization, we decided to include here the complete fatty acid profiles of all the 18 cheese categories analyzed. National and international nutritional databases make available data on cheese fatty acids averaged from many heterogeneous sources [38,39]. Due to the very detailed fatty acid profiles obtained and the large number of cheese categories considered, the results have been reported here in several tables comparing, in each table, all the cheese categories by individual groups of fatty acids.

### 3.2. Cheese, Fatty Acid Saturation, and Carbon Chain Length

Starting with the level of saturation of the fatty acids (Table 2), it is worth noting that the three categories of cheeses made from milk produced on Alpine summer farms (Malga fresco RTC, Malga vecchio RTC, and Trentino di Malga) had the lowest (*p* < 0.05) proportions of saturated fatty acids (SFAs, about 60–61% of the total fatty acid content), and the highest (*p* < 0.05) contents of monounsaturated fatty acids (MUFAs, 33–34%) and polyunsaturated fatty acids (PUFAs, 6–7%). Among the other cheese categories, only Morlacco del Grappa RTC had a lower than average SFA content (63.67%; *p* < 0.05), and only goat cheeses had a higher than average proportion (70.7%; *p* < 0.05) of SFA, while goat cheeses and Monte Veronese PDO cheeses had lower than average (24–26%; *p* < 0.05) proportions of MUFAs. Monte Veronese PDO cheese had a higher than average (5.75%; *p* < 0.05) proportion of PUFAs, while five other categories had lower than average (4.4–4.7%; *p* < 0.05) proportions of PUFAs (Table 2).

The lengths of the carbon chains of the fatty acids (Table 2) confirmed the distinctiveness of the three Malga cheese categories, which had the lowest proportions of short-chain fatty acids (23–24%; *p* < 0.05) and medium-chain (27–28%; *p* < 0.05) fatty acids, along with goat cheeses (28.25%; *p* < 0.05), in contrast to Mozzarella TSG + other soft *pasta filata* cheeses, Flavored cheeses, and Semi-hard cheeses (≈33%; *p* < 0.05); however, they had the highest proportions of long-chain fatty acids (≈49%; *p* < 0.05), along with Morlacco del Grappa RTC (45%; *p* < 0.05), in contrast to the Flavored cheeses and Goat cheeses (37–39%; *p* < 0.05).

### 3.3. Proportions of Even-, Odd-, and Branched-Chain SFAs

The differences among the cheese categories in terms of the sum of all linear even-chain SFAs are very similar to those observed for all SFAs, the large majority of which are linear even-chain fatty acids (Table 3). The same pattern was also observed for all the individual short- and medium-chain fatty acids, except butyric acid (C4:0). In fact, the three Malga cheese categories and Morlacco del Grappa RTC generally had the lowest proportions of these fatty acids (*p* < 0.05), whereas the goat cheeses and Piave PDO + other hard cheeses had the highest (*p* < 0.05). The butyric acid content was found to be higher than average in Montasio PDO, Monte Veronese PDO, and Morlacco del Grappa RTC cheeses (3.1–3.3%; *p* < 0.05) and lower in Treated rind cheeses (2.70%; *p* < 0.05) and goat cheeses (2.11%; *p* < 0.05). The patterns of individual long-chain linear even SFAs tended to be the opposite of those for short- and medium-chain SFAs, with the Malga categories, and sometimes Monte Veronese PDO and Morlacco del Grappa RTC, having the highest contents (*p* < 0.05).

The sum of all the linear odd-chain SFAs did not vary much among the cheese categories, whereas there were some significant differences in individual fatty acids between categories, except C19:0 (Table 4). These patterns differed for the various individual fatty acids, with the three Malga categories tending to have lower proportions of C7:0, C11:0, and C13:0 (*p* < 0.05) and higher proportions of C17:0 (*p* < 0.05).

The differences among cheese categories in terms of the sum and the proportions of individual branched-chain SFAs tended to have a more homogeneous pattern (except for C19:0 *iso*), with the Malga, Monte Veronese PDO, and Morlacco del Grappa RTC categories having the highest values (*p* < 0.05) and the goat cheeses the lowest values (*p* < 0.05) (Table 5).

### 3.4. Proportions of MUFAs

The differences among the cheese types in terms of the relative proportions of individual MUFAs are summarized in Table 6 and Table 7. Here, too, the goat cheeses and the three categories of Malga cheeses were the more distinctive. The goat cheeses presented significant (*p* < 0.05) deviations from the average for 13 of the 22 individual MUFAs and the sum of the MUFA isomers quantified. Compared with all the other cheese categories, out of the 22 MUFAs and sums of MUFA isomers analyzed, the goat cheeses had the lowest proportions (*p* < 0.05) of eight MUFAs (C14:1 *cis*-9, C16:1 *cis*-9, ΣC17:1, C18:1 *cis*-9, C18:1 *cis*-11, ΣC19:1, C20:1 *cis*-8, and ΣC20:1) and the highest proportions (*p* < 0.05) of four MUFAs (ΣC14:1, ΣC15:1, ΣC16:1, and C20:1 *cis*-11) and the sums of the MUFA isomers.

Among the bovine cheeses, the three Malga cheese categories (made from cows’ milk produced on Alpine summer farms) differed significantly (*p* < 0.05) from the other cheese categories in several of the individual MUFAs and the sums of the MUFA isomers (16 out of 22 for Malga fresco RTC, 14 for Malga vecchio RTC, and 15 for Trentino di Malga). These cheese categories often had the lowest proportions (*p* < 0.05) of short-chain MUFAs (C ≤ 14) and the highest proportions of mid- and long-chain MUFAs (C ≥ 15), with the exception of C16:1 *cis*-9, C18:1 *cis*-12, the sum of C19:1 isomers, and the sum of C22:1 isomers.

The other 14 cheese categories were much less variable in terms of their proportions of MUFAs, and only 1 to 5 individual MUFAs and the sums of the MUFA isomers deviated from the general mean (*p* < 0.05).

### 3.5. Proportions of CLA Isomers, Omega-3, Omega-6, and Other PUFAs

The first group of nutritionally important PUFAs comprises the conjugated linoleic acid (CLA) isomers, summarized in Table 8. The three Malga cheese categories had the highest proportions of the sum of all the CLA isomers (1.4–1.7; *p* < 0.05) as well as C18:2 *cis*-9, *cis*-11, C18:2 *cis*-9, *trans*-11, and the sum of the remaining CLA isomers, except C18:2 *cis*-11, *trans*-13. Other cheese categories of mountain origin presented significantly higher proportions of CLA isomers than the general means: Monte Veronese PDO for C18:2 *cis*-9, *trans*-11 (0.986%; *p* < 0.05); Montasio PDO for C18:2 *cis*-11, *trans*-13 (0.059%; *p* < 0.05); and Morlacco del Grappa RTC for Σothers CLA (0.113%; *p* < 0.05).

Lower than average (*p* < 0.05) proportions of CLA isomers (Σ CLA and C18:2 *cis*-9, *trans*-11) were found in the two *pasta filata* categories (Provolone PDO + other hard *pasta filata* cheeses and Mozzarella TSG + other soft *pasta filata* cheeses), the Grana Padano cheeses (C18:2 *cis*-9, *cis*-11), the Flavored cheeses, and Treated rind cheeses (Σ CLA and C18:2 *cis*-9, *trans*-11).

The proportions of the sum of all omega-3 PUFAs and the sum of the major individual omega-3 PUFAs are summarized in Table 9. The highest proportions (*p* < 0.05) of these PUFA were found in the three Malga categories in particular and in other categories of cheeses of mountain origin (Monte Veronese PDO, Piave PDO + other hard cheeses, and Morlacco del Grappa RTC), and in only one omega-3 PUFA in the goat cheeses. The lowest proportions (*p* < 0.05) were found in the two *pasta filata* categories of cheeses, the fresh cheeses (Casatella Trevigiana PDO + other fresh cheeses and Caciotta and Latteria), the Flavored cheeses, and the Treated rind cheeses.

The pattern was very different in the case of omega-6 MUFAs, as summarized in Table 10. The three Malga cheese categories and the goat cheeses had the lowest proportions (*p* < 0.05), while the PDO cheeses had the highest proportions (*p* < 0.05).

The proportions of the other PUFAs are summarized in Table 11. Significant differences in these were more sporadic, with some notable exceptions regarding the goat cheeses and the Malga categories of cheeses.

### 3.6. Variability in the Detailed Fatty Acid Profiles of Different Cheese Types

In this study, we found wide variation in the detailed fatty acid profiles of the 258 different cheeses analyzed. Type of cheese was a very important source of variation. In fact, only five of the 88 individual fatty acids, sums of isomers of specific fatty acids, and fatty acid groups were not significantly affected by cheese type. Our analyses show that these differences are not randomly distributed among cheese types, but instead, there are several associations (co-variations) among the different fatty acids and the different categories of cheeses. Unfortunately, the large numbers of fatty acids and cheese types make it difficult to identify the main drivers of these associations by simply examining the LSMs of the fatty acid proportions reported in the results tables.

**Figure 1:**
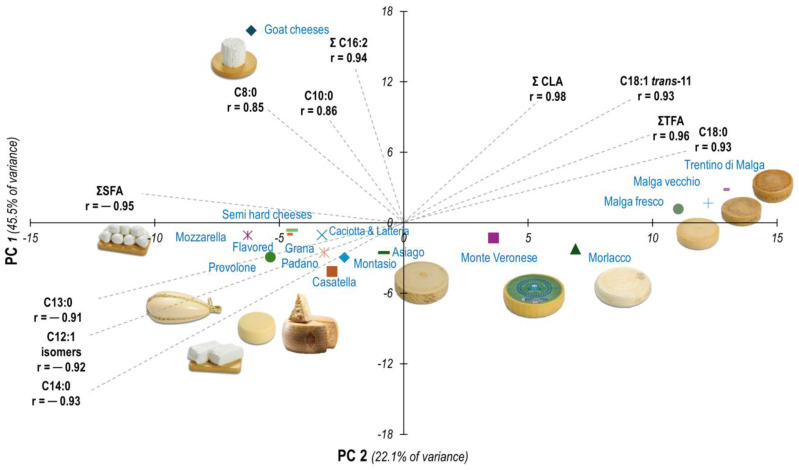
Principal component analysis of the detailed fatty acid profiles of 18 cheese categories.

Further clarity may be gained by analyzing the data with a multivariate statistical technique, such as principal component analysis, and representing the results graphically (Figure 1).

The large number of associations between the fatty acids and the cheese categories is demonstrated by the fact that the first principal component alone explains 45.5% of all the variability, while the second explains a further 22.1%. Therefore, the first two principal components represented in the figure combined explain about two-thirds of all the variability. As information on farming systems, feeding practices, animal breeds, milk composition, etc., is not available, it is not possible to discriminate between the effects of these factors and those related to cheese-making technologies; this requires specific research based on an ad hoc experimental design. In this study, the effect of cheese type should be considered as the result of the combined effects of the area of production, farm characteristics, attitude, and, consequently, milk properties.

It is evident that goat cheeses are very distinct from all the bovine cheeses. Among the latter, the Malga cheese categories, made from milk produced on Alpine summer farms, clustered together and were very easily distinguished from all the other categories. The other cheeses of mountain origin, particularly Morlacco del Grappa and Monte Veronese, also tended to be distinct from the cheeses produced on the plains. Lastly, less evident clustering was obtained when comparing the two *pasta filata* cheese categories with the remaining categories. No multivariate analysis of cheese types has been reported.

### 3.7. Detailed Fatty Acid Profiles of Caprine and Bovine Cheeses

Goat milk has certain particular characteristics compared with other dairy species, especially in the coagulation, curd-firming, and syneresis processes [40,41].

It is well known that goat milk also has a different fatty acid profile to cow’s milk [42] and other dairy species [43], and that what mainly distinguishes caprine milk is the relative abundance of short-chain, even, SFAs, except for the shortest, C4:0 [28,44,45]. This well-established knowledge can be seen in the common names of these fatty acids: caproic acid (C6:0), caprylic acid (C8:0), and capric acid (C10:0). *Capra* is the Latin word for a goat. In contrast, goat cheeses, like goat milk, have the lowest contents of butyric acid (C4:0), palmitic acid (C16:0), and stearic acid (C18:0), and of branched-chain SFAs, often assumed to be indicators of microbial fermentation in the rumen. The second principal component illustrated in Figure 1 (representing 22.1% of all the variability) seems mainly related to the species of the dairy animal, with the goat cheeses having a very high (positive) score and all the cows’ cheese categories having small to negative scores.

### 3.8. Detailed Fatty Acid Profiles of Cheeses Produced on Summer Highland Farms

Malga cheeses are often produced in small traditional dairies annexed to temporary summer farms on Alpine pastures and are very distinctive mountain cheeses [46,47,48,49].

The presence of several MUFAs and PUFAs in the fresh forage is mainly responsible for the distinctive fatty acid profile of milk produced by grazing cows, and this is, of course, reflected in the fatty acid profile of the cheese produced from that milk.

Particularly interesting is the presence and activity of rumenic acid (C18:2, *cis*-9, *trans*-11), the main CLA in forage, milk, and cheese, and of its precursor vaccenic acid (C18:1 trans-11) [50]. CLAs play an important role in human nutrition due to their function in preventing inflammation [51].

The high content of CLA isomers in fresh forage, milk, and cheese is probably the most important marker of dairy products produced in grazing farming systems; however, CLA isomers, particularly C18:2, *trans*-10, *cis*-12, are also responsible for a significant alteration in lipid metabolism, with reduced synthesis of many other fatty acids [52,53] and in non-bovine dairy species [54,55]. Similar proportions of CLAs in the fats of several cheeses obtained from milk produced by non-grazing cows were found in other studies [30], but more CLA isomers are contained in cheeses made from the milk of cows grazing in highland pastures than from the milk of cows kept indoors [44].

### 3.9. Detailed Fatty Acid Profiles of Cheeses Produced in the Mountains and on the Plains

The majority of the PDO and RTC cheeses sampled for this study claim in their designations to have their origins in the mountains (Asiago, Montasio, Monte Veronese, Morlacco del Grappa), and consumers associate these cheeses with traditional mountain dairy farming. However, the production areas defined by the PDO regulations of these cheeses also include the foothills and plains below these mountains [56,57], where the majority of these cheeses are produced in intensive farming systems. This is evident from the clustering of cheese groups illustrated in Figure 1. The first principal component (explaining 45.5% of all fatty acid profile variability) is clearly related to the environment/dairy-farming system where the milk for cheese-making is produced. In fact, the highest (positive) scores were obtained by the three Malga cheese categories. The *Pasta filata* cheese categories had the lowest (negative) scores, and the majority of the commercial cheese groups (mainly from intensive farms on the plains) had small negative scores. It is interesting that the PDO and RTC cheeses of mountain origin differ from each other, and the Asiago and Montasio PDO cheeses are at the margins of the cluster of commercial cheeses, with slightly negative scores, whereas the Monte Veronese PDO and Morlacco del Grappa RTC cheeses (local cheeses with smaller production levels) are in an intermediate position between the Asiago and Montasio cheeses and the Malga cheeses. The detailed fatty acid profiles seem to attest to the classification of Monte Veronese and Morlacco del Grappa cheeses as mountain cheeses, whereas the Asiago and Montasio cheeses are probably very variable according to the area and factory of production.

### 3.10. Detailed Fatty Acid Profiles of Other Commercial Cheese Categories

Among the other cheese categories, those with the lowest scores for the first principal component are the two categories of *pasta filata* cheeses. The majority of these cheeses come from the plains, like the majority of the remaining groups. Although the primary driver of variability between cheese categories is the length of ripening [58,59,60], *pasta filata* cheeses are characterized by very short (the category including Mozzarella TSG) or medium ripening lengths (the category including Provolone PDO). Therefore, if they are not differentiated from the other categories in terms of the origin of the milk or the length of ripening, it is probable that the main factor differentiating them is the cheese-making technology and, in particular, the high temperature of the curd stretching phase and the alterations it induces in the physical, chemical, and (especially) microbiological properties of *pasta filata* cheeses [61,62,63].

The remaining groups clustered together despite the large variability in milk processing and the addition of other substances (spices, herbs, smoke, etc.), meaning that these techniques are not responsible for the considerable modifications to their fatty acid profiles. It is worth noting that the Grana category also clustered with the other groups, even though these cheeses are the heaviest (35 kg per wheel, on average) and ripened for the longest period (up to 36 months) among all the categories tested [17]. On the other hand, Grana Padano is produced on the plains from milk from intensive farms that keep mainly Holstein cows fed on total mixed rations with corn silage and concentrates [61,64,65]. It is also produced in mountain areas in traditional farming systems [66].

## 4. Conclusions

We characterized 18 different categories of cheeses using highly detailed fatty acid profiles. Fatty acids present in small concentrations are increasingly interesting because of their possible effects on human metabolism (bioactive compounds) and health and as a source of volatile organic compounds responsible for some sensory properties. The results clearly showed that, aside from the distinctive FA profiles of goat cheeses (more short-chain FAs and fewer MUFAs), the three categories of artisanal cheeses produced on temporary summer farms on Alpine pastures were very different from the other cheese categories in terms of their much higher CLAs and omega-3 contents. Two categories of cheeses from permanent farms in the mountains were intermediate, and two other categories of cheeses originating in the mountains, but now produced mainly on the plains, were not distinguishable from the other cheese categories. Also, the technology used for producing *pasta filata* cheeses seems to cause some modification to the fatty acid profile. The very detailed fatty acid profile of many cheese categories found in this study could become a reference for further technological, sensorial, or nutritional research.

## Figures and Tables

**Table 1:** Main characteristics of 18 categories under protected designation of origin (PDO, 7 categories with 17 cheese types) denomination, traditional specialty guaranteed (TSG, 1 cheese type), Veneto regional traditional designation (RTC, 4 cheese types), and 17 other commercial cheese types.

Cheese Category	Cheese Types N	Cheeses sampled N	Milk Fat Content ^1^	Heat Treat ^2^	Cheese Ripening Time (Months)	Cheese Paste Firmness	Notes
Asiago PDO cheeses	4	20	variable	yes/no	1 to 24	soft to hard	mountain name
Casatella PDO + other fresh cheeses	2	20	full fat	yes	<1	very fresh	no rind cheeses
Grana Padano PDO	2	10	skim	no	12 to 36	very hard	from plains
Montasio PDO cheeses	3	12	full fat	yes/no	2 to 18	soft to hard	mountain name
Monte Veronese PDO	3	14	variable	yes/no	1 to 24	soft to hard	mountain name
Piave PDO + other hard cheeses	2	9	skim	yes/no	1 to 24	soft to hard	mountain river name
Provolone PDO + pasta filata	3	9	full fat	yes	2 to 12	soft to firm	stretched hard cheeses
Mozzarella TSG + pasta filata	2	7	full fat	yes	<1 to 3	fresh to soft	fresh stretched curd
Formaggio inbriago RTC	1	7	full fat	yes/no	2–6	firm	in marc/must/wine
Morlacco del Grappa RTC	1	7	skim	yes/no	1–3	soft	mountain name
Malga fresco RTC	1	42	skim	no	2–6	firm	from Alpine pastures
Malga vecchio RTC	1	19	skim	no	12 to 24	very hard	from Alpine pastures
Trentino di Malga	1	24	skim	no	4 to 24	firm to hard	from Alpine pastures
Caciotta and Latteria	2	12	full fat	yes	1–4	semi-soft	from plains
Flavored cheeses	5	14	full fat	yes	var.	variable	with spices, herbs, smoke
Semi-hard cheeses	1	8	variable	yes/no	3–6	firm	from plains
Treated rind	2	10	full fat	yes	1–6	semi-hard	washed or moldy rind
Goat cheese	2	14	full fat	yes	<1 to 6	fresh to firm	acid or rennet coag.

^1^ Milk fat content: full fat = milk not skimmed; skim = partly skimmed milk; variable = varies according to single cheese type and dairy. ^2^ Heat treatment of milk: yes/no = varies according to cheese type and dairy; PDO: protected designation of origin according to the European Union [21]; TSG: traditional specialty guaranteed cheese according to the European Union [21]; RTC: regional traditional cheese according to the Veneto Region.

**Table 2:** Contents of total solids and lipids (g/100 g) of the 258 individual cheeses analyzed and the proportions of fatty acids (% of total content) according to their saturation and carbon-chain length: descriptive statistics, F-values, and significance of the effects of cheese categories (*** = *p* < 0.001) and their least squares means (LSMs) (red bold values are inferior (*p* < 0.05) and green bold values are superior (*p* < 0.05) to the general average value of all groups).

	Composition		FA Saturation			FA Chain Length		
	Solids	Lipids	SFA	MUFA	PUFA	C ≤ 15	C16	C ≥ 17
**Descriptive statistics:**								
mean	59.7	28.94	65.42	29.08	5.50	26.52	30.30	43.17
SD	10.0	5.00	4.53	3.90	0.97	3.38	3.12	5.65
min	40.4	12.17	55.04	10.36	3.54	17.28	24.61	25.97
max	71.4	42.46	83.54	39.50	7.95	40.09	39.06	54.79
**F-value**	30.3 ***	14.9 ***	25.0 ***	21.2 ***	16.3 ***	28.3 ***	19.5 ***	24.5 ***
**LSMs of Cheese categories:**								
Asiago PDO cheeses	**66.5**	30.94	67.00	27.81	5.19	26.86	31.46	41.58
Casatella PDO + other fresh cheeses	**43.7**	24.63	66.69	28.39	4.92	26.84	32.36	40.79
Grana Padano PDO	**68.0**	27.31	67.87	27.04	5.09	27.75	31.84	40.41
Montasio PDO cheeses	**66.6**	31.81	68.21	26.85	4.94	27.93	31.91	40.14
Monte Veronese PDO	**65.0**	31.12	68.10	**26.15**	**5.75**	27.62	31.40	40.98
Piave PDO + other hard cheeses	**71.4**	**34.54**	68.44	26.35	5.20	28.76	32.06	39.15
Provolone PDO + pasta filata	60.8	27.44	68.23	27.09	**4.69**	27.36	32.90	39.74
Mozzarella TSG + pasta filata	**40.4**	**19.93**	68.53	27.00	**4.47**	28.03	**33.48**	38.54
Formaggio inbriago RTC	**65.9**	31.38	66.88	27.87	5.25	27.01	31.77	41.16
Morlacco del Grappa RTC	**47.3**	**23.14**	**63.67**	30.40	5.93	25.08	29.86	**45.05**
Malga fresco RTC	61.3	29.69	**60.73**	**33.02**	**6.25**	**23.45**	**27.47**	**49.05**
Malga vecchio RTC	**66.2**	30.55	**60.15**	**33.30**	**6.54**	**23.18**	**27.28**	**49.58**
Trentino di Malga	**65.5**	29.81	**60.78**	**32.79**	**6.43**	**23.74**	**27.24**	**49.02**
Caciotta and Latteria	**54.7**	27.53	67.15	28.21	**4.64**	27.27	32.50	40.15
Flavored cheeses	**64.3**	32.02	68.39	26.92	**4.69**	28.03	**33.14**	**38.84**
Semi-hard cheeses	**68.3**	**33.63**	68.85	26.41	4.73	28.47	**33.07**	38.44
Treated rind	**53.9**	27.11	68.61	27.00	**4.38**	28.08	33.07	38.85
Goat cheese	**44.0**	**19.83**	**70.70**	**23.81**	5.49	**34.45**	**28.25**	**37.35**

**Table 3:** Content of even, saturated, linear-chain fatty acids and their sums (ΣESL-FA) expressed as % of the sum of all fatty acids in the 258 individual cheeses analyzed: descriptive statistics, *p*-values, and significance of the effects of cheese categories (*** = *p* < 0.001) and their LSMs (red bold values are inferior (*p* < 0.05) and green bold values are superior (*p* < 0.05) to the general average value of all groups).

	ΣESL-FA	C4:0	C6:0	C8:0	C10:0	C12:0	C14:0	C16:0	C18:0	C20:0	C22:0	C24:0
**Descriptive statistics:**												
mean	60.48	2.88	1.9	1.22	2.80	2.94	10.48	27.92	9.91	0.23	0.10	0.10
SD	8.87	0.31	0.18	0.35	1.58	0.68	1.25	3.17	1.22	0.05	0.04	0.04
min	41.99	1.84	1.47	0.77	1.35	1.55	6.63	22.03	6.24	0.09	0.01	0.01
max	91.57	4.32	2.73	2.73	10.49	5.94	14.14	37.02	13.29	0.4	0.22	0.31
**F-value**	27.0 ***	19.5 ***	12.7 ***	83.0 ***	124 ***	26.2 ***	16.4 ***	20.9 ***	10.2 ***	8.0 ***	8.1 ***	4.0 ***
**LSMs of Cheese categories:**												
Asiago PDO cheeses	62.11	2.88	1.86	1.16	2.57	3.06	10.93	29.42	9.81	0.25	0.10	0.09
Casatella PDO + other fresh cheeses	61.81	3.04	1.90	1.14	**2.46**	2.94	10.80	30.03	9.07	0.22	0.10	0.09
Grana Padano PDO	63.03	2.91	1.94	1.21	2.74	3.24	11.22	29.68	9.70	0.23	0.08	0.08
Montasio PDO cheeses	63.31	**3.08**	1.98	1.22	2.72	3.20	11.33	29.78	9.61	0.23	0.09	0.11
Monte Veronese PDO	63.05	**3.33**	1.99	1.19	2.56	2.99	11.12	29.11	**10.28**	**0.25**	0.11	0.11
Piave PDO + other hard cheeses	63.58	2.89	**2.05**	**1.38**	3.18	**3.39**	11.50	29.68	9.14	**0.19**	0.09	0.10
Provolone PDO + pasta filata	63.42	2.85	1.91	1.20	2.66	3.17	11.15	30.61	9.52	0.21	0.07	**0.06**
Mozzarella TSG + pasta filata	63.83	2.89	1.99	1.26	2.76	3.32	11.31	**30.99**	9.00	**0.18**	**0.06**	0.08
Formaggio inbriago RTC	61.67	3.04	1.90	1.15	2.52	2.99	10.81	29.53	9.31	0.21	0.10	0.10
Morlacco del Grappa RTC	**58.60**	**3.30**	1.81	**1.06**	**2.16**	**2.56**	**9.79**	27.52	9.93	**0.29**	**0.14**	0.09
Malga fresco RTC	**55.66**	2.92	**1.78**	**1.03**	**2.02**	**2.32**	**9.23**	**24.97**	**10.90**	0.24	**0.12**	**0.12**
Malga vecchio RTC	**55.10**	2.86	**1.73**	**1.01**	**1.96**	**2.28**	**9.23**	**24.71**	**10.85**	0.24	**0.12**	**0.13**
Malga Trentino	**55.51**	2.87	**1.80**	**1.05**	**2.08**	**2.37**	**9.34**	**24.60**	**10.90**	0.22	**0.13**	**0.13**
Caciotta and latteria	62.38	2.84	1.93	1.20	2.59	3.15	11.15	30.00	9.15	**0.19**	0.09	0.08
Flavored cheeses	63.51	2.79	1.98	1.25	2.75	3.32	**11.43**	**30.58**	9.11	**0.18**	**0.06**	0.07
Semi-hard cheeses	**64.20**	2.71	2.02	1.30	2.89	3.46	**11.67**	**30.71**	9.07	**0.18**	0.08	0.10
Treated rind	63.74	**2.70**	1.97	1.31	3.09	3.31	11.30	30.57	9.12	0.20	0.08	0.09
Goat cheese	**66.26**	**2.11**	**2.22**	**2.49**	**8.75**	**4.39**	10.95	**26.21**	**8.65**	**0.28**	0.12	0.09

**Table 4:** Contents of odd, saturated, linear-chain fatty acids and of their sums (ΣOSL-FA) expressed as % of the sum of all fatty acids in the 258 individual cheeses analyzed: descriptive statistics, *p*-values, and significance of the effects of cheese categories (*** = *p* < 0.001; * = *p* < 0.05) and their LSMs (red bold values are inferior (*p* < 0.05) and green bold values are superior (*p* < 0.05) to the general average value of all groups).

	ΣOSL-FA	C5:0	C7:0	C9:0	C11:0	C13:0	C15:0	C17:0	C19:0	C21:0
**Descriptive statistics:**										
mean	2.48	0.12	0.13	0.08	0.08	0.13	1.15	0.67	0.09	0.04
SD	0.40	0.02	0.05	0.03	0.02	0.03	0.13	0.08	0.03	0.02
min	1.43	0.08	0.02	0.01	0.03	0.05	0.69	0.52	0.01	0.01
max	3.69	0.19	0.25	0.16	0.16	0.19	1.53	0.93	0.15	0.13
**F-value**	1.8 *	6.9 ***	6.3 ***	11.7 ***	18.6 ***	13.1 ***	3.2 ***	4.7 ***	1.6	3.3 ***
**LSMs of Cheese categories:**										
Asiago PDO cheeses	2.45	0.12	0.15	**0.05**	0.08	0.13	1.15	0.65	0.09	0.04
Casatella PDO + other fresh cheeses	2.50	0.13	**0.18**	**0.06**	0.08	0.14	1.15	0.66	0.08	0.03
Grana Padano PDO	2.55	0.14	**0.18**	0.06	0.10	0.15	1.16	0.65	0.09	0.03
Montasio PDO cheeses	2.46	0.13	0.17	**0.06**	0.09	0.14	1.15	0.64	0.09	0.02
Monte Veronese PDO	2.38	0.13	**0.09**	**0.05**	0.08	0.13	1.19	**0.71**	0.10	0.04
Piave PDO + other hard cheeses	2.49	0.11	0.14	0.08	0.09	0.15	1.17	0.62	0.08	0.04
Provolone PDO + pasta filata	2.53	0.14	**0.18**	0.09	0.10	0.15	1.15	0.63	0.08	0.03
Mozzarella TSG + pasta filata	2.54	0.13	0.16	**0.10**	0.10	0.16	1.19	0.60	0.08	0.03
Formaggio inbriago RTC	2.65	0.13	0.18	0.08	0.09	0.13	1.24	0.70	0.09	0.04
Morlacco del Grappa RTC	2.44	**0.15**	0.11	0.07	0.08	0.12	1.16	0.70	0.10	0.04
Malga fresco RTC	2.40	0.11	**0.11**	0.08	**0.06**	**0.11**	1.10	**0.71**	0.09	0.04
Malga vecchio RTC	2.37	0.11	**0.09**	0.08	**0.05**	**0.11**	1.09	**0.71**	0.10	0.04
Malga Trentino	2.48	0.12	0.12	0.08	**0.06**	**0.12**	1.17	**0.71**	0.08	0.04
Caciotta and latteria	2.44	**0.11**	0.13	0.08	0.08	0.15	1.18	0.64	0.07	0.02
Flavored cheeses	2.59	**0.15**	0.14	**0.10**	0.09	**0.16**	1.23	0.64	0.07	0.03
Semi-hard cheeses	2.48	**0.11**	0.13	0.09	0.09	**0.16**	1.20	0.60	0.08	0.02
Treated rind	2.49	0.11	0.13	0.08	0.09	0.15	1.20	0.63	0.08	0.02
Goat cheese	2.36	**0.11**	0.13	**0.12**	**0.12**	0.14	**0.97**	0.63	0.08	**0.07**

**Table 5:** Contents of saturated branched-chain fatty acids and of their sums (ΣSB-FA) expressed as % of the sum of all fatty acids in the 258 individual cheeses analyzed: descriptive statistics, *p*-values, and significance of the effects of cheese categories (*** = *p* < 0.001; ** = *p* < 0.01) and their LSMs (red bold values are inferior (*p* < 0.05) and green bold values are superior (*p* < 0.05) to the general average value of all groups).

	ΣSB-FA	C13:0 *iso*	C14:0 *iso*	C15:0 *iso*	C15:0 *anteiso*	C16:0 *iso*	C17:0 *iso*	C17:0 *anteiso*	C18:0 *iso*	C19:0 *iso*
**Descriptive statistics:**										
mean	2.48	0.07	0.18	0.30	0.57	0.34	0.41	0.52	0.09	0.02
SD	0.40	0.02	0.04	0.07	0.10	0.05	0.07	0.07	0.03	0.01
min	1.43	0.02	0.10	0.13	0.32	0.22	0.20	0.24	0.02	0.01
max	3.69	0.12	0.30	0.59	0.91	0.53	0.70	0.74	0.24	0.07
**F-value**	9.5 ***	6.8 ***	4.6 ***	7.2 ***	8.7 ***	2.5 **	11.5 ***	3.3 ***	4.5 ***	1.5
**LSMs of Cheese categories:**										
Asiago PDO cheeses	2.44	0.06	0.18	0.29	0.55	0.34	0.38	0.51	**0.11**	0.01
Casatella PDO + other fresh cheeses	2.38	0.07	0.17	0.28	0.54	0.34	0.36	0.51	0.10	0.02
Grana Padano PDO	2.29	0.06	0.17	0.27	0.52	0.34	**0.34**	0.51	0.08	0.01
Montasio PDO cheeses	2.44	0.06	0.19	0.29	0.55	0.37	0.38	0.52	0.09	0.02
Monte Veronese PDO	**2.67**	0.07	**0.20**	**0.33**	**0.63**	0.36	**0.44**	**0.56**	0.10	0.02
Piave PDO + other hard cheeses	2.37	0.06	0.18	0.29	0.55	0.35	0.39	0.47	0.07	0.02
Provolone PDO + pasta filata	2.28	**0.05**	0.16	0.27	0.51	0.31	0.36	0.51	0.09	0.02
Mozzarella TSG + pasta filata	2.15	0.06	0.16	**0.23**	0.50	0.30	0.34	0.49	0.07	0.02
Formaggio inbriago RTC	2.56	0.06	0.18	0.32	0.57	0.36	0.41	0.54	0.11	0.02
Morlacco del Grappa RTC	2.64	0.08	**0.21**	0.34	0.61	**0.39**	0.43	0.53	0.09	0.02
Malga fresco RTC	**2.68**	**0.08**	**0.20**	**0.34**	**0.62**	0.35	**0.46**	0.52	**0.11**	0.02
Malga vecchio RTC	**2.69**	**0.08**	0.19	**0.34**	**0.62**	0.35	**0.46**	0.54	0.10	0.02
Malga Trentino	**2.80**	**0.08**	**0.20**	**0.36**	**0.65**	0.33	**0.49**	**0.57**	**0.11**	0.02
Caciotta and latteria	2.32	0.06	0.16	0.26	0.54	0.33	0.37	0.51	0.08	0.02
Flavored cheeses	2.28	0.06	0.16	0.26	0.50	0.34	0.37	0.51	0.07	0.02
Semi-hard cheeses	**2.17**	0.06	0.16	0.26	0.51	0.31	**0.33**	0.48	**0.06**	0.03
Treated rind	2.38	0.06	0.17	0.28	0.54	0.35	0.36	0.54	0.08	0.02
Goat cheese	**2.00**	**0.05**	**0.14**	**0.23**	**0.40**	**0.30**	0.37	**0.44**	**0.06**	0.02

**Table 6:** Contents of major short- and medium-length carbon-chain mono-unsaturated fatty acids and sums of other isomers expressed as % of the sum of all fatty acids in the 258 individual cheeses: descriptive statistics, *p*-values, and significance of the effects of cheese categories (*** = *p* < 0.001) and their LSMs (red bold values are inferior (*p* < 0.05) and green bold values are superior (*p* < 0.05) to general average value of all groups).

	C10:1	ΣC12:1	C14:1*cis*-9	ΣotherC14:1	ΣC15:1	C16:1*cis*-9	C16:1 *t*-9	ΣotherC16:1	ΣC17:1
**Descriptive statistics:**									
mean	0.29	0.21	0.82	0.21	0.09	1.34	0.17	0.20	0.36
SD	0.04	0.04	0.21	0.06	0.04	0.26	0.10	0.15	0.07
min	0.17	0.10	0.15	0.09	0.01	0.44	0.02	0.01	0.24
max	0.39	0.33	1.17	0.51	0.19	2.07	0.44	0.67	0.69
**F-value**	10.4 ***	10.5 ***	36.1 ***	16.0 ***	5.9 ***	20.6 ***	19.4 ***	25.4 ***	15.9 ***
**LSMs of Cheese categories:**									
Asiago PDO cheeses	0.31	0.23	**0.96**	0.21	0.08	1.45	**0.10**	**0.03**	0.32
Casatella PDO + other fresh cheeses	0.32	0.24	**1.03**	0.22	0.08	**1.58**	0.10	**0.02**	0.34
Grana Padano PDO	0.31	0.24	**0.96**	0.23	0.08	1.42	**0.09**	**0.03**	0.30
Montasio PDO cheeses	0.31	0.23	0.92	0.22	0.08	1.38	0.10	**0.03**	**0.29**
Monte Veronese PDO	0.31	0.21	0.90	**0.19**	0.10	1.38	0.18	**0.06**	0.36
Piave PDO + other hard cheeses	0.30	0.22	0.83	0.24	0.08	1.29	0.15	**0.28**	0.31
Provolone PDO + pasta filata	0.31	0.23	0.95	0.23	**0.04**	1.48	0.09	0.12	0.34
Mozzarella TSG + pasta filata	0.32	0.24	**0.98**	0.25	0.08	**1.53**	0.09	0.23	0.33
Formaggio inbriago RTC	0.30	0.23	0.93	0.23	0.10	1.45	0.14	**0.02**	0.34
Morlacco del Grappa RTC	0.29	0.21	0.88	0.22	0.10	1.35	0.20	**0.05**	0.35
Malga fresco RTC	**0.25**	**0.18**	**0.74**	**0.17**	**0.11**	1.31	**0.24**	0.20	**0.42**
Malga vecchio RTC	**0.25**	**0.18**	**0.77**	**0.17**	**0.10**	1.29	**0.27**	0.21	**0.41**
Malga Trentino	**0.25**	**0.18**	**0.67**	**0.17**	**0.11**	**1.27**	**0.30**	**0.27**	**0.47**
Caciotta and latteria	0.31	0.23	**0.96**	0.22	0.07	1.50	0.11	**0.32**	0.36
Flavored cheeses	0.31	0.24	0.93	0.26	0.06	1.47	0.11	**0.33**	0.34
Semi-hard cheeses	0.31	0.24	0.92	0.24	0.06	1.44	0.09	**0.31**	0.32
Treated rind	0.31	0.23	0.90	0.24	0.06	1.43	0.10	**0.32**	0.35
Goat cheese	**0.27**	0.22	**0.24**	**0.34**	**0.11**	**0.62**	0.15	**0.43**	**0.29**

**Table 7:** Contents of major long-length carbon-chain mono-unsaturated fatty acids and sums of other isomers expressed as % of the sum of all fatty acids of the 258 cheeses: descriptive statistics, *p*-values, and significance of the effect of cheese categories (*** = *p* < 0.001; ** = *p* < 0.01; * = *p* < 0.05) and their LSMs (red bold values are inferior (*p* < 0.05) and green bold values are superior (*p* < 0.05) to general average value of all groups).

	C18:1 *cis*-9	C18:1 *cis*-11	C18:1 *t*-11	C18:1 *cis*-12	C18:1 *cis*-13	C18:1 *cis*-15	C18:1 *cis*-16	ΣC18:1-*t*	ΣC19:1	C20:1 *cis*-8	C20:1 *cis*-11	ΣC20:1	ΣC22:1
**Descriptive statistics:**													
mean	20.24	0.56	2.48	0.29	0.08	0.12	0.08	1.03	0.21	0.17	0.07	0.24	0.03
SD	3.00	0.08	1.34	0.09	0.05	0.03	0.02	0.87	0.04	0.05	0.03	0.06	0.01
min	1.07	0.29	0.09	0.1	0.01	0.04	0.03	0.19	0.08	0.01	0.01	0.03	0.01
max	29.81	0.79	5.84	0.55	0.47	0.33	0.15	5.97	0.6	0.29	0.21	0.38	0.08
**F-value**	15.5 ***	9.9 ***	3.7 ***	10.7 ***	12.9 ***	2.5 **	4.1 ***	17.1 ***	3.1 ***	4.1 ***	2.1 **	10.8 ***	1.9 *
**Cheese categories LSMs:**													
Asiago PDO cheeses	19.93	0.55	2.15	0.32	0.05	0.12	0.08	0.52	0.20	0.17	0.08	0.24	0.02
Casatella PDO + other fresh cheeses	20.21	0.57	1.82	0.33	0.04	0.10	0.08	0.61	0.21	**0.20**	0.08	**0.28**	0.03
Grana Padano PDO	19.07	0.57	1.91	**0.40**	0.04	0.13	0.10	1.33	0.23	0.19	0.07	0.23	0.02
Montasio PDO cheeses	19.06	0.55	**1.00**	0.34	0.09	0.11	0.07	**1.71**	0.23	0.20	0.08	0.27	0.02
Monte Veronese PDO	**17.29**	**0.49**	2.17	**0.23**	0.05	0.11	0.08	1.36	0.21	0.19	0.07	0.26	0.03
Piave PDO + other hard cheeses	18.23	0.53	1.97	0.33	0.08	0.11	0.08	0.75	0.21	0.15	0.06	0.20	0.02
Provolone PDO + pasta filata	19.08	0.58	1.46	**0.37**	0.07	0.11	0.08	0.91	0.23	0.18	0.08	0.25	0.01
Mozzarella TSG + pasta filata	18.93	0.56	1.38	0.36	0.07	0.11	0.07	0.90	0.20	0.16	0.07	0.22	0.01
Formaggio inbriago RTC	19.36	0.55	2.05	0.29	0.06	0.12	0.08	0.94	0.21	0.19	0.08	0.27	0.02
Morlacco del Grappa RTC	21.19	0.59	2.77	**0.24**	0.07	0.14	0.09	0.90	0.20	**0.23**	0.08	**0.31**	0.04
Malga fresco RTC	**22.92**	**0.59**	**3.35**	**0.24**	**0.10**	**0.14**	0.09	**1.67**	0.21	**0.19**	0.08	**0.27**	0.03
Malga vecchio RTC	**22.84**	0.59	**4.00**	**0.24**	0.07	**0.14**	**0.09**	0.90	0.21	**0.20**	0.09	**0.28**	0.03
Malga Trentino	**22.11**	**0.59**	**4.20**	**0.19**	0.09	**0.14**	**0.09**	0.92	0.20	0.18	0.06	0.25	0.02
Caciotta and latteria	19.93	0.56	1.88	0.30	0.09	0.10	0.07	0.57	0.22	0.18	0.06	0.24	0.03
Flavored cheeses	18.62	**0.59**	1.57	0.35	0.09	0.11	0.07	0.88	0.21	0.16	0.06	0.22	0.02
Semi-hard cheeses	18.26	0.51	1.81	0.39	0.09	0.11	0.08	0.74	0.22	0.15	**0.03**	**0.19**	0.02
Treated rind	19.06	0.52	1.55	0.32	0.09	**0.09**	**0.06**	0.72	**0.25**	0.16	0.06	0.22	0.02
13Goat cheese	**17.33**	**0.39**	1.71	0.27	0.04	0.10	0.07	0.78	**0.16**	**0.04**	**0.09**	**0.12**	0.02

**Table 8:** Sum of all conjugated linoleic acids (CLAs) and contents of the major CLA isomers and sum of the other isomers expressed as % of the sum of all fatty acids in the 258 individual cheeses analyzed: descriptive statistics, *p*-values, and significance of the effects of cheese categories (*** = *p* < 0.001) and their LSMs (red bold values are inferior (*p* < 0.05) and green bold values are superior (*p* < 0.05) to the general average value of all groups).

	Σ CLA	C18:2 *cis*-9, *cis*-11	C18:2 *cis*-9, *trans*-11	C18:2 *cis*-11, *trans*-13	Σ Others CLA
**Descriptive statistics:**					
mean	0.98	0.08	0.90	0.02	0.07
SD	0.51	0.06	0.45	0.03	0.04
min	0.31	0.01	0.29	0.01	0.01
max	2.25	0.24	2.04	0.47	0.28
**F-value**	8.9 ***	4.5 ***	10.1 ***	4.8 ***	1.0
**LSMs of Cheese categories:**					
Asiago PDO cheeses	0.706	0.036	0.661	0.015	0.065
Casatella PDO + other fresh cheeses	0.711	0.033	0.654	0.024	0.040
Grana Padano PDO	0.612	**0.023**	0.575	0.018	0.061
Montasio PDO cheeses	0.655	0.031	0.622	**0.059**	0.062
Monte Veronese PDO	1.055	0.056	**0.986**	0.020	0.064
Piave PDO + other hard cheeses	0.727	0.050	0.679	0.016	0.041
Provolone PDO + pasta filata	**0.526**	0.021	0.498	0.018	0.037
Mozzarella TSG + pasta filata	**0.514**	0.022	**0.491**	0.018	0.047
Formaggio inbriago RTC	0.875	0.065	0.786	0.024	0.063
Morlacco del Grappa RTC	1.124	0.083	1.047	0.025	**0.113**
Malga fresco RTC	**1.399**	**0.129**	**1.263**	0.025	**0.089**
Malga vecchio RTC	**1.584**	**0.132**	**1.426**	0.028	**0.107**
Malga Trentino	**1.627**	**0.144**	**1.464**	0.027	**0.119**
Caciotta and latteria	0.687	0.043	0.632	0.019	0.050
Flavored cheeses	0.580	0.053	**0.543**	0.016	0.063
Semi-hard cheeses	0.574	0.051	0.550	0.019	0.033
Treated rind	**0.575**	0.026	**0.541**	0.026	0.042
Goat cheese	0.705	0.043	0.645	0.028	0.044

**Table 9:** Sum of all the omega-3 fatty acids and contents of the major omega-3 fatty acids expressed as % of the sum of all fatty acids in the 258 individual cheeses analyzed: descriptive statistics, *p*-values, and significance of the effect of cheese categories (*** = *p* < 0.001) and their LSMs (red bold values are inferior (*p* < 0.05) and green bold values are superior (*p* < 0.05) to the general average value of all groups).

	ΣΩ 3	C16:3 Ω3 HTA	C18:3 Ω3 ALA	C20:3 Ω3 ETE	C20:4 Ω3 ETA	C20:5 Ω3 EPA	C22:5 Ω3 DPA
**Descriptive statistics:**							
mean	0.91	0.03	0.62	0.02	0.07	0.08	0.13
SD	0.28	0.02	0.21	0.01	0.02	0.02	0.03
min	0.44	0.01	0.29	0.01	0.01	0.03	0.02
max	1.66	0.12	1.27	0.06	0.13	0.16	0.29
**F-value**	11.5 ***	3.2 ***	11.6 ***	3.5 ***	10.3 ***	5.1 ***	4.7 ***
**LSMs of Cheese categories:**							
Asiago PDO cheeses	0.882	0.013	0.603	0.014	0.059	0.077	0.131
Casatella PDO + other fresh cheeses	0.664	0.015	**0.445**	0.015	0.045	**0.064**	0.102
Grana Padano PDO	0.834	0.016	0.582	0.013	0.051	0.076	0.117
Montasio PDO cheeses	0.766	0.016	0.510	0.016	0.053	0.077	0.119
Monte Veronese PDO	**0.990**	0.018	0.654	0.022	0.070	**0.100**	**0.152**
Piave PDO + other hard cheeses	0.893	**0.045**	0.613	0.021	0.054	0.081	0.131
Provolone PDO + pasta filata	**0.658**	0.017	0.435	0.015	0.046	0.066	0.101
Mozzarella TSG + pasta filata	**0.621**	0.018	**0.411**	-	0.048	0.067	0.095
Formaggio inbriago RTC	0.931	0.015	0.619	0.021	0.074	0.086	0.137
Morlacco del Grappa RTC	0.965	0.037	0.677	0.023	**0.082**	0.089	0.102
Malga fresco RTC	**1.072**	0.028	**0.757**	0.024	**0.081**	0.086	0.132
Malga vecchio RTC	**1.118**	0.033	**0.780**	0.031	**0.081**	**0.093**	0.136
Malga Trentino	**1.207**	**0.038**	**0.843**	0.029	**0.090**	**0.097**	**0.154**
Caciotta and latteria	0.650	0.019	**0.420**	0.019	0.049	0.066	0.106
Flavored cheeses	0.704	0.022	**0.445**	0.021	0.057	0.076	0.121
Semi-hard cheeses	0.716	0.034	0.478	0.035	0.046	0.074	0.114
Treated rind	**0.652**	0.020	**0.417**	0.013	0.045	0.069	0.116
Goat cheese	0.867	0.032	0.615	0.020	**0.023**	0.083	**0.150**

**Table 10:** Sum of all the omega-6 fatty acids, content of the major omega-6 fatty acids expressed as % of the sum of all fatty acids, and ratio between omega-6 and omega-3 fatty acids in the 258 individual cheeses analyzed: descriptive statistics, *p*-values, and significance of the effects of cheese categories (*** = *p* < 0.001; * = *p* < 0.05) and their LSMs (red bold values are inferior (*p* < 0.05) and green bold values are superior (*p* < 0.05) to the general average value of all groups).

	ΣΩ6	C20:2 Ω6 EDA	C18:3 Ω6 GLA	C20:3 Ω6 DGLA	C20:4 Ω6 AA	C22:4 Ω6 Adrenic	Ω6/Ω3
**Descriptive statistics:**							
mean	0.34	0.04	0.06	0.13	0.17	0.03	0.43
SD	0.08	0.02	0.01	0.03	0.03	0.02	0.20
min	0.17	0.01	0.02	0.01	0.09	0.01	0.13
max	0.55	0.09	0.11	0.20	0.27	0.10	1.23
**F-value**	17.5 ***	3.9 ***	3.0 ***	23.0 ***	12.1 ***	2.1 *	13.7 ***
**LSMs of Cheese categories:**							
Asiago PDO cheeses	**0.426**	0.052	0.062	**0.152**	0.184	0.043	0.51
Casatella PDO + other fresh cheeses	**0.443**	0.053	0.064	**0.150**	**0.195**	0.050	**0.71**
Grana Padano PDO	**0.428**	0.049	0.063	0.155	0.190	0.043	0.53
Montasio PDO cheeses	**0.425**	0.057	0.066	**0.151**	0.190	0.037	0.57
Monte Veronese PDO	**0.407**	0.054	0.067	**0.146**	0.188	0.032	0.42
Piave PDO + other hard cheeses	0.349	0.045	0.066	0.133	0.177	0.037	0.46
Provolone PDO + pasta filata	0.403	0.044	0.069	**0.148**	0.197	0.034	**0.62**
Mozzarella TSG + pasta filata	0.367	0.030	0.060	0.136	0.171	0.038	0.61
Formaggio inbriago RTC	0.380	0.049	0.065	0.141	0.178	0.033	0.46
Morlacco del Grappa RTC	0.373	0.041	0.067	0.139	0.180	0.043	0.46
Malga fresco RTC	**0.290**	0.036	0.060	**0.113**	**0.144**	**0.026**	**0.29**
Malga vecchio RTC	**0.282**	0.046	0.065	**0.111**	**0.145**	**0.021**	**0.27**
Malga Trentino	**0.252**	0.016	**0.047**	**0.105**	**0.131**	0.029	**0.22**
Caciotta and latteria	0.342	0.023	0.057	0.133	0.168	0.032	0.55
Flavored cheeses	0.351	-	0.062	0.136	0.178	0.037	0.54
Semi-hard cheeses	0.350	-	0.058	0.139	0.169	0.047	0.55
Treated rind	0.319	0.021	0.060	0.120	0.170	0.029	0.51
Goat cheese	**0.267**	0.039	0.053	**0.042**	**0.192**	0.028	**0.33**

**Table 11:** Contents of other major polyunsaturated fatty acids expressed as % of the sum of all fatty acids in the 258 individual cheeses analyzed: descriptive statistics, *p*-values, and significance of the effects of cheese categories (*** = *p* < 0.001) and their LSMs (red bold values are inferior (*p* < 0.05) and green bold values are superior (*p* < 0.05) to the general average value of all groups).

	ΣC16:2	C18:2 *cis*-9, *cis*-12	C18:2 *cis*-9, *cis*-15	C18:2 *cis*-9, *trans*-12	C18:2 *cis*-9, *trans*-13	C18:2 *trans*-8, *cis*-13	C18:2 *trans*-9, *cis*-12	C18:2 *trans*-11, *cis*-15	C18:2 *trans*-9, *trans*-12
**Descriptive statistics:**									
mean	0.03	1.91	0.06	0.16	0.27	0.21	0.07	0.32	0.09
SD	0.02	0.35	0.02	0.04	0.04	0.07	0.18	0.26	0.07
min	0.01	1.12	0.01	0.01	0.18	0.05	0.01	0.02	0.01
max	0.09	3.21	0.11	0.33	0.49	0.44	2.32	2.13	0.29
**F-value**	4.4 ***	4.9 ***	1.6	22.5 ***	1.0	14.8 ***	9.0 ***	23.6 ***	7.8 ***
**LSMs of Cheese categories:**									
Asiago PDO cheeses	0.012	2.027	0.048	**0.093**	0.252	**0.267**	**0.262**	0.300	0.042
Casatella PDO + other fresh cheeses	0.012	2.002	0.060	0.167	0.251	0.243	0.089	0.189	0.016
Grana Padano PDO	0.014	2.079	0.051	0.160	0.276	0.245	0.058	0.378	0.013
Montasio PDO cheeses	0.014	1.971	0.058	0.158	0.243	0.246	0.056	0.180	0.048
Monte Veronese PDO	0.013	1.982	0.051	0.140	0.267	**0.291**	0.048	0.292	0.099
Piave PDO + other hard cheeses	0.039	1.984	0.049	0.169	0.267	0.211	0.077	0.438	0.017
Provolone PDO + pasta filata	0.013	1.992	0.060	0.141	0.257	0.247	0.018	0.174	0.023
Mozzarella TSG + pasta filata	0.016	1.855	0.059	0.155	0.261	0.192	0.022	0.161	0.015
Formaggio inbriago RTC	0.021	1.806	0.061	**0.110**	0.256	0.239	0.134	0.196	0.070
Morlacco del Grappa RTC	0.025	1.938	0.068	0.142	0.284	0.244	0.055	0.369	0.086
Malga fresco RTC	0.031	1.853	0.065	0.157	0.292	0.190	0.077	**0.458**	**0.135**
Malga vecchio RTC	0.026	1.842	0.064	0.175	0.306	0.182	0.059	**0.464**	**0.119**
Malga Trentino	0.027	**1.584**	0.056	0.168	0.295	**0.151**	0.072	**0.515**	**0.168**
Caciotta and latteria	0.025	1.831	0.051	0.154	0.242	0.209	0.019	0.192	0.023
Flavored cheeses	0.018	1.862	0.055	0.163	0.256	0.191	0.025	0.196	0.084
Semi-hard cheeses	0.021	1.949	0.052	0.178	0.255	0.187	0.091	0.199	0.016
Treated rind	0.021	1.752	0.056	0.161	0.236	0.186	0.035	0.172	0.030
Goat cheese	**0.071**	**2.458**	0.046	**0.212**	0.290	**0.105**	0.067	0.199	0.024

## Data Availability

The datasets presented in this article are not readily available because the data are part of an ongoing study.

## References

[1] Rodríguez-Alcalá L.M., Castro-Gómez M.P., Pimentel L.L., Fontecha J. (2017). Milk Fat Components with Potential Anticancer Activity—A Review. Biosci. Rep..

[2] Dehghan M., Mente A., Zhang X., Swaminathan S., Li W., Mohan V., Iqbal R., Kumar R., Wentzel-Viljoen E., Rosengren A. (2017). Associations of Fats and Carbohydrate Intake with Cardiovascular Disease and Mortality in 18 Countries from Five Continents (PURE): A Prospective Cohort Study. Lancet.

[3] Chowdhury R., Warnakula S., Kunutsor S., Krowe F., Ward H.A., Johnson L., Franco O.H., Butterworth A.S., Forouhl N.G., Thompson S.G. (2014). Association of Dietary, Circulating, and Supplement Fatty Acids With Coronary Risk. Ann. Intern. Med..

[4] Astrup A., Magkos F., Bier D.M., Brenna J.T., de Oliveira Otto M.C., Hill J.O., King J.C., Mente A., Ordovas J.M., Volek J.S. (2020). Saturated Fats and Health: A Reassessment and Proposal for Food-Based Recommendations: JACC State-of-the-Art Review. J. Am. Coll. Cardiol..

[5] Heileson J.L. (2020). Dietary Saturated Fat and Heart Disease: A Narrative Review. Nutr. Rev..

[6] Nestel P.J., Mori T.A. (2022). Dairy Foods: Is Its Cardiovascular Risk Profile Changing?. Curr. Atheroscler. Rep..

[7] Feeney E.L., Lamichhane P., Sheehan J.J. (2021). The Cheese Matrix: Understanding the Impact of Cheese Structure on Aspects of Cardiovascular Health—A Food Science and a Human Nutrition Perspective. Int. J. Dairy Technol..

[8] International Daily Federation (2021). Cheese and Varieties Part II: Cheese Styles.

[9] Law B.A., Tamine A.Y. (2010). Technology of Cheesemaking.

[10] Fox P.F., Guinee T.P., Cogan T.M., McSweeney P.L.H. (2016). Fundamentals of Cheese Science.

[11] Cattani M., Mantovani R., Schiavon S., Bittante G., Bailoni L. (2014). Recovery of N-3 Polyunsaturated Fatty Acids and Conjugated Linoleic Acids in Ripened Cheese Obtained from Milk of Cows Fed Different Levels of Extruded Flaxseed. J. Dairy Sci..

[12] Adamska A., Rasińska E., Rutkowska J., Antoniewska A. (2017). Fatty Acid Profile of Commercial Camembert- and Brie-Type Cheeses Available on the Polish Market. CYTA J. Food.

[13] Di Trana A., Di Rosa A.R., Addis M., Fiori M., Di Grigoli A., Morittu V.M., Spina A.A., Claps S., Chiofalo V., Licitra G. (2022). The Quality of Five Natural, Historical Italian Cheeses Produced in Different Months: Gross Composition, Fat-Soluble Vitamins, Fatty Acids, Total Phenols, Antioxidant Capacity, and Health Index. Animals.

[14] Margalho L.P., Kamimura B.A., Pimentel T.C., Balthazar C.F., Araujo J.V.A., Silva R., Conte-Junior C.A., Raices R.S.L., Cruz A.G., Sant’Ana A.S. (2021). A Large Survey of the Fatty Acid Profile and Gross Composition of Brazilian Artisanal Cheeses. J. Food Compos. Anal..

[15] Ochoa-Flores A.A., Hernández-Becerra J.A., Velázquez-Martínez J.R., Piña-Gutiérrez J.M., Hernández-Castellano L.E., Toro-Mujica P., Chay-Canul A.J., Vargas-Bello-Pérez E. (2021). Chemical and Fatty Acid Composition of Manchego Type and Panela Cheeses Manufactured from Either Hair Sheep Milk or Cow Milk. J. Dairy Sci..

[16] Subaşi K., Tirpanci Sivri G., Taşan M., Öksüz Ö. (2022). Determination of the Physicochemical Properties and Fatty Acid Composition of Some Cheese Types with Geographical Indication in Thrace Region. Yuz. Yil Univ. J. Agric. Sci..

[17] Bittante G., Amalfitano N., Cipolat-Gotet C., Lombardi A., Stocco G., Tagliapietra F. (2022). Major Causes of Variation of External Appearance, Chemical Composition, Texture, and Color Traits of 37 Categories of Cheeses. Foods.

[18] Bittante G., Amalfitano N., Ferragina A., Lombardi A., Tagliapietra F. (2024). Interrelationships among Physical and Chemical Traits of Cheese: Explanatory Latent Factors and Clustering of 37 Categories of Cheeses. J. Dairy Sci..

[19] Stocco G., Cipolat-Gotet C., Ferragina A., Berzaghi P., Bittante G. (2019). Accuracy and Biases in Predicting the Chemical and Physical Traits of Many Types of Cheeses Using Different Visible and Near-Infrared Spectroscopic Techniques and Spectrum Intervals. J. Dairy Sci..

[20] Bittante G., Patel N., Cecchinato A., Berzaghi P. (2022). Invited Review: A Comprehensive Review of Visible and near-Infrared Spectroscopy for Predicting the Chemical Composition of Cheese. J. Dairy Sci..

[21] European Union Enhancing Quality Schemes for Agricultural Products and Foodstuffs—Regulation (EU) No 1151/2012 on Quality Schemes for Agricultural Products and Foodstuffs. https://eur-lex.europa.eu/legal-content/EN/ALL/?uri=LEGISSUM%3A0905_2.

[22] Dias C., Mendes L. (2018). Protected Designation of Origin (PDO), Protected Geographical Indication (PGI) and Traditional Speciality Guaranteed (TSG): A Bibiliometric Analysis. Food Res. Int..

[23] (2001). ISO Milk Products.

[24] Schiavon S., Pellattiero E., Cecchinato A., Tagliapietra F., Dannenberger D., Nuernberg K., Nuernberg G., Bittante G. (2016). The Influence of Different Sample Preparation Procedures on the Determination of Fatty Acid Profiles of Beef Subcutaneous Fat, Liver and Muscle by Gas Chromatography. J. Food Compos. Anal..

[25] Schiavon S., Cesaro G., Cecchinato A., Cipolat-Gotet C., Tagliapietra F., Bittante G. (2016). The Influence of Dietary Nitrogen Reduction and Conjugated Linoleic Acid Supply to Dairy Cows on Fatty Acids in Milk and Their Transfer to Ripened Cheese. J. Dairy Sci..

[26] Cobos A., de Oliveira Filho E.F., Miranda M., Ferreiro T., López-Alonso M., Díaz O., Herrero-Latorre C. (2024). Chemometric Characterization of the Fatty Acid and Trace Element Profiles of Organic and Conventional Galician Cheeses. Appl. Food Res..

[27] Collins Y.F., McSweeney P.L.H., Wilkinson M.G. (2003). Lipolysis and Free Fatty Acid Catabolism in Cheese: A Review of Current Knowledge. Int. Dairy J..

[28] de la Fuente M.A., Fontecha J., Juárez M. (1993). Fatty Acid Composition of the Triglyceride and Free Fatty Acid Fractions in Different Cows-, Ewes- and Goats-Milk Cheeses. Z. Lebensm. Unters. Forsch..

[29] Buccioni A., Minieri S., Conte G., Benvenuti D., Pezzati A., Antongiovanni M., Rapaccini S., Mele M. (2012). Changes in Conjugated Linoleic Acid and C18:1 Isomers Profile during the Ripening of Pecorino Toscano Cheese Produced with Raw Milk. Ital. J. Anim. Sci..

[30] Cicognini F.M., Rossi F., Sigolo S., Gallo A., Prandini A. (2014). Conjugated Linoleic Acid Isomer (Cis9,Trans11 and Trans10,Cis12) Content in Cheeses from Italian Large-Scale Retail Trade. Int. Dairy J..

[31] Lobos-Ortega I., Hernández-Jiménez M., González-Martín M.I., Hernández-Hierro J.M., Revilla I., Vivar-Quintana A.M. (2021). Study of Polyunsaturated Fatty Acids in Cheeses Using Near-Infrared Spectroscopy: Influence of Milk from Different Ruminant Species. Food Anal. Methods.

[32] Hyötyläinen T., Kallio M., Lehtonen M., Lintonen S., Peräjoki P., Jussila M., Riekkola M.L. (2004). Comprehensive Two-Dimensional Gas Chromatography in the Analysis of Dietary Fatty Acids. J. Sep. Sci..

[33] Zeng A.X., Chin S.T., Marriott P.J. (2013). Integrated Multidimensional and Comprehensive 2D GC Analysis of Fatty Acid Methyl Esters. J. Sep. Sci..

[34] Teng C., Ren R., Liu Z., Wang J., Shi S., Kang Y.E., Koo B.S., Lu W., Shan Y. (2024). C15:0 and C17:0 Partially Mediate the Association of Milk and Dairy Products with Bladder Cancer Risk. J. Dairy Sci..

[35] Marilley L., Casey M.G. (2004). Flavours of Cheese Products: Metabolic Pathways, Analytical Tools and Identification of Producing Strains. Int. J. Food Microbiol..

[36] Bergamaschi M., Bittante G. (2017). Detailed Fatty Acid Profile of Milk, Cheese, Ricotta and by Products, from Cows Grazing Summer Highland Pastures. J. Dairy Res..

[37] Pegolo S., Stocco G., Mele M., Schiavon S., Bittante G., Cecchinato A. (2017). Factors Affecting Variations in the Detailed Fatty Acid Profile of Mediterranean Buffalo Milk Determined by 2-Dimensional Gas Chromatography. J. Dairy Sci..

[38] USDA (2018). Composition of Foods: Raw, Processed, Prepared USDA National Nutrient Database for Standard Reference, Legacy.

[39] USDA FoodData Central. https://fdc.nal.usda.gov/.

[40] Stocco G., Pazzola M., Dettori M.L., Paschino P., Bittante G., Vacca G.M. (2018). Effect of Composition on Coagulation, Curd Firming, and Syneresis of Goat Milk. J. Dairy Sci..

[41] Vacca G.M., Stocco G., Dettori M.L., Summer A., Cipolat-Gotet C., Bittante G., Pazzola M. (2018). Cheese Yield, Cheesemaking Efficiency, and Daily Production of 6 Breeds of Goats. J. Dairy Sci..

[42] Yang J., Zheng N., Wang J., Yang Y. (2018). Comparative Milk Fatty Acid Analysis of Different Dairy Species. Int. J. Dairy Technol..

[43] Wang F., Chen M., Luo R., Huang G., Wu X., Zheng N., Zhang Y., Wang J. (2022). Fatty Acid Profiles of Milk from Holstein Cows, Jersey Cows, Buffalos, Yaks, Humans, Goats, Camels, and Donkeys Based on Gas Chromatography–Mass Spectrometry. J. Dairy Sci..

[44] Prandini A., Sigolo S., Tansini G., Brogna N., Piva G. (2007). Different Level of Conjugated Linoleic Acid (CLA) in Dairy Products from Italy. J. Food Compos. Anal..

[45] Ali A.H., Khalifa S.A., Gan R.Y., Shah N., Ayyash M. (2023). Fatty Acids, Lipid Quality Parameters, and Amino Acid Profiles of Unripened and Ripened Cheeses Produced from Different Milk Sources. J. Food Compos. Anal..

[46] Buchin S., Martin B., Dupont D., Bornard A., Achilleos C. (1999). Influence of the Composition of Alpine Highland Pasture on the Chemical, Rheological and Sensory Properties of Cheese. J. Dairy Res..

[47] Bovolenta S., Corazzin M., Saccà E., Gasperi F., Biasioli F., Ventura W. (2009). Performance and Cheese Quality of Brown Cows Grazing on Mountain Pasture Fed Two Different Levels of Supplementation. Livest. Sci..

[48] Romanzin A., Corazzin M., Piasentier E., Bovolenta S. (2013). Effect of Rearing System (Mountain Pasture vs. Indoor) of Simmental Cows on Milk Composition and Montasio Cheese Characteristics. J. Dairy Res..

[49] Bergamaschi M., Cipolat-Gotet C., Stocco G., Valorz C., Bazzoli I., Sturaro E., Ramanzin M., Bittante G. (2016). Cheesemaking in Highland Pastures: Milk Technological Properties, Cream, Cheese and Ricotta Yields, Milk Nutrients Recovery, and Products Composition. J. Dairy Sci..

[50] Gong M., Hu Y., Wei W., Jin Q., Wang X. (2019). Production of Conjugated Fatty Acids: A Review of Recent Advances. Biotechnol. Adv..

[51] Penedo L.A., Nunes J.C., Gama M.A.Ô.S., Leite P.E.C., Quirico-Santos T.F., Torres A.G. (2013). Intake of Butter Naturally Enriched with Cis9,Trans11 Conjugated Linoleic Acid Reduces Systemic Inflammatory Mediators in Healthy Young Adults. J. Nutr. Biochem..

[52] Kelsey J.A., Corl B.A., Collier R.J., Bauman D.E. (2003). The Effect of Breed, Parity, and Stage of Lactation on Conjugated Linoleic Acid (CLA) in Milk Fat from Dairy Cows. J. Dairy Sci..

[53] Maxin G., Glasser F., Hurtaud C., Peyraud J.L., Rulquin H. (2011). Combined Effects of Trans-10,Cis-12 Conjugated Linoleic Acid, Propionate, and Acetate on Milk Fat Yield and Composition in Dairy Cows. J. Dairy Sci..

[54] Ghazal S., Berthelot V., Friggens N.C., Schmidely P. (2012). Influence of a Supplement Containing Conjugated Linoleic Acid on Dairy Performance, Milk Fatty Acid Composition, and Adipose Tissue Reactivity to Lipolytic Challenge in Mid-Lactation Goats. J. Dairy Sci..

[55] Oliveira D.E., Gama M.A.S., Fernandes D., Tedeschi L.O., Bauman D.E. (2012). An Unprotected Conjugated Linoleic Acid Supplement Decreases Milk Production and Secretion of Milk Components in Grazing Dairy Ewes. J. Dairy Sci..

[56] Kędzierska-Matysek M., Barłowska J., Wolanciuk A., Litwińczuk Z. (2018). Physicochemical, Mechanical and Sensory Properties of Long-Ripened Polish and Italian Cheeses and Their Content of Selected Minerals. J. Elem..

[57] European-Union Regulation (EU) No 1151/2012 of the European Parliament and of the Council of 21 November 2012 on Quality Schemes for Agricultural Products and Foodstuffs. https://eur-lex.europa.eu/eli/reg/2012/1151/oj/eng.

[58] Trmčić A., Ralyea R., Meunier-Goddik L., Donnelly C., Glass K., D’Amico D., Meredith E., Kehler M., Tranchina N., McCue C. (2017). Consensus Categorization of Cheese Based on Water Activity and PH—A Rational Approach to Systemizing Cheese Diversity. J. Dairy Sci..

[59] Lamichhane P., Kelly A.L., Sheehan J.J. (2018). Symposium Review: Structure-Function Relationships in Cheese. J. Dairy Sci..

[60] Putranto A., Woo M.W., Selomulya C., Chen X.D. (2018). An Accurate Account of Mass Loss during Cheese Ripening Described Using the Reaction Engineering Approach (REA)-Based Model. Int. J. Food Sci. Technol..

[61] Gobbetti M., Neviani E., Fox P., Varanini G.M. (2018). The Cheeses of Italy: Science and Technology.

[62] Ah J., Tagalpallewar G.P. (2017). Functional Properties of Mozzarella Cheese for Its End Use Application. J. Food Sci. Technol..

[63] Fusco V., Chieffi D., De Angelis M. (2022). Invited Review: Fresh Pasta Filata Cheeses: Composition, Role, and Evolution of the Microbiota in Their Quality and Safety. J. Dairy Sci..

[64] Mucchetti G., Pugliese A., Paciulli M. (2017). Characteristics of Some Important Italian Cheeses. Mediterranean Foods.

[65] da Silva Duarte V., Lombardi A., Corich V., Giacomini A. (2022). Assessment of the Microbiological Origin of Blowing Defects in Grana Padano Protected Designation of Origin Cheese. J. Dairy Sci..

[66] Bittante G., Cologna N., Cecchinato A., De Marchi M., Penasa M., Tiezzi F., Endrizzi I., Gasperi F. (2011). Monitoring of Sensory Attributes Used in the Quality Payment System of Trentingrana Cheese. J. Dairy Sci..

